# Elevated atrial blood stasis in paroxysmal atrial fibrillation during sinus rhythm: a patient-specific computational fluid dynamics study

**DOI:** 10.3389/fcvm.2023.1219021

**Published:** 2023-08-15

**Authors:** Sophia Bäck, Iulia Skoda, Jonas Lantz, Lilian Henriksson, Lars O. Karlsson, Anders Persson, Carl-Johan Carlhäll, Tino Ebbers

**Affiliations:** ^1^ Unit of Cardiovascular Sciences, Department of Health, Medicine and Caring Sciences, Linköping University, Linköping, Sweden; ^2^ Center for Medical Image Science and Visualization (CMIV), Linköping University, Linköping, Sweden; ^3^ Department of Cardiology in Linköping, and Department of Health, Medicine and Caring Sciences, Linköping University, Linköping, Sweden; ^4^ Department of Radiology, and Department of Health, Medicine and Caring Sciences, Linköping University, Linköping, Sweden; ^5^ Department of Clinical Physiology in Linköping, and Department of Health, Medicine and Caring Sciences, Linköping University, Linköping, Sweden

**Keywords:** atrial fibrillation, computational fluid dynamics, left atrial appendage, computed tomography, atrial cardiomyopathy, stroke

## Abstract

**Introduction:**

Atrial fibrillation (AF) is associated with an increased risk of stroke, often caused by thrombi that form in the left atrium (LA), and especially in the left atrial appendage (LAA). The underlying mechanism is not fully understood but is thought to be related to stagnant blood flow, which might be present despite sinus rhythm. However, measuring blood flow and stasis in the LAA is challenging due to its small size and low velocities. We aimed to compare the blood flow and stasis in the left atrium of paroxysmal AF patients with controls using computational fluid dynamics (CFD) simulations.

**Methods:**

The CFD simulations were based on time-resolved computed tomography including the patient-specific cardiac motion. The pipeline allowed for analysis of 21 patients with paroxysmal AF and 8 controls. Stasis was estimated by computing the blood residence time.

**Results and Discussion:**

Residence time was elevated in the AF group (*p* < 0.001). Linear regression analysis revealed that stasis was strongest associated with LA ejection ratio (*p* < 0.001, *R*^2 ^= 0.68) and the ratio of LA volume and left ventricular stroke volume (*p* < 0.001, *R*^2 ^= 0.81). Stroke risk due to LA thrombi could already be elevated in AF patients during sinus rhythm. In the future, patient specific CFD simulations may add to the assessment of this risk and support diagnosis and treatment.

## Introduction

Atrial fibrillation (AF) is the most common cardiac arrhythmia and is strongly associated with increased risk of stroke. While the underlying mechanisms are incompletely understood, the risk of stroke in AF is largely attributed to prothrombotic blood flow stasis occurring in the atrium when the normal chamber contraction and periodicity are lost to the chaotic rhythm. Paroxysmal AF is of particular interest for understanding the multifactorial mechanisms behind stroke in AF, since the increased stroke risk is present even though the patients are in organized sinus rhythm most of the time (1). As with persistent AF, paroxysmal AF patients are often prescribed anticoagulation medications to reduce stroke risk at the cost of increased risk of bleeding (2). However, patient-specific stroke risk assessment to date is generally based on demographic and comorbid conditions rather than pathophysiological mechanisms. In the clinic, stroke risk is commonly assessed though the CHA_2_DS_2_-VASc score (3), which takes the occurrence of cardiovascular diseases such as heart failure, hypertension, diabetes, stroke and vascular diseases, as well as age and sex into account. It is a rough measure that was found to identify patients at low risk, but only modestly identifies patients at high risk (4). Stroke risk assessment that also takes the patient specific cardiac condition into account could lead to more patient specific treatment planning, including more optimized use of anticoagulants, possibly leading to reduced incidence of cardiac emboli related stroke as well as bleedings.

The thrombi causing cardioembolic strokes in AF patients are believed to originate in the left atrial appendage (LAA) (5), a highly trabeculated extension of the left atrium (LA) which varies largely in size and morphology between individuals (6, 7). A recent debate revolves around whether the risk of stroke is attributable to fibrillating motion of the atrium, or whether an underlying atrial cardiomyopathy could cause both AF and stroke (8). In that case, stroke risk would be elevated even during sinus rhythm. Much of the focus on atrial cardiomyopathy has been on alterations in the atrial wall itself including inflammation, and less on its effects on atrial function and flow field (9). The interplay between atrial geometry, function, and blood flow on the stroke risk of AF patients therefore requires further study.

To investigate these features, detailed information on atrial shape and motion is needed. In the setting of clinical AF, transesophageal echocardiography (TEE) is commonly used to detect LAA thrombi but this is invasive, often requires patient sedation, and cannot define the full 3D blood flow in the LA and LAA (10). 4D flow magnetic resonance imaging (MRI) has been used to evaluate the atrial blood flow field (11), and has shown decreased blood flow velocity in AF patients during sinus rhythm compared to controls (12). 4D flow MRI has a relatively coarse spatial resolution and low sensitivity to lower velocities, however, and therefore cannot be used to study small regions such as the LAA, or low-velocity regions which are the most prone to stasis. Therefore, neither TEE nor 4D flow MRI are optimal for fully examining the function of the LA and LAA. In recent years, the use of cardiac CT has greatly expanded since it offers a high spatial resolution and can clearly distinguish the cardiac wall due to iodine-based contrast in the blood pool. The radiation exposure from CT can be substantially reduced with advanced dose modulation (13). Although CT does not provide inherent motion information, we previously showed that it allows for the extraction of the endocardial wall motion and that flow rates calculated from those data agree well with measurements from 4D flow MRI (14). Thus, time-resolved cardiac CT is an excellent tool to investigate the left atrial shape and function. However, it does not directly provide information on the velocity field and stasis risk in the heart, as it cannot measure flow information.

To bridge this gap, the flow in the heart can be computed using computational fluid dynamics (CFD) based on cardiac CT, with results similar to 4D flow MRI (15, 16). Beside the flow field, CFD also allows for stasis assessment (17). Stasis can be estimated by analyzing the blood washout (18, 19) or by computing a passive scalar representing the blood residence time (20). However, CFD simulations are very computationally expensive and often require a lot of manual preprocessing, limiting most studies to a small number of patients (20–24). In 2021, two studies were published with larger patient cohorts; Sanatkhani et al. computed the flow field in 16 AF patients (18) and Mill et al. analyzed 52 AF patients (25). However, both studies did not take the patient specific wall motion into account, hindering the analysis of the atrial function and its effects on stasis. García-Villalba et al. compared the flow field in the LA simulated with rigid and moving walls in 6 patients and found that the wall motion influences the flow field substantially (20), which was also observed by Vella et al. (26). Computational fluid dynamics has been shown to be a valuable tool in stasis assessment in the LA in small patient groups. Applying it to larger patient cohorts while including patient specific geometry and motion would extend our understanding of atrial cardiomyopathy, its effect on atrial blood flow, and ultimately the increased stroke risk in paroxysmal AF patients.

In the current study, we aimed to compare the blood flow and stasis in the left atrium of paroxysmal AF patients with controls using CFD simulations. For this, we developed a CFD pipeline using time-resolved CT data and accounting for patient-specific atrial wall movement to characterize in unprecedented detail the hemodynamics and blood stasis in both AF patients during sinus rhythm and in controls. The pipeline allows for analysis of a comparably large patient cohort, bringing the method closer to clinical practice. The comparison of atrial hemodynamics and key morphological and functional cardiac descriptors between the two groups provides a deeper understanding of the mechanisms resulting in a higher stroke risk in paroxysmal AF. The devised setup could further be used to estimate the thrombus risk on a patient specific level and guide clinicians in personalized stroke prevention for AF patients.

## Materials and methods

### Study participants

The study included 21 individuals with paroxysmal AF and 8 individuals without AF, recruited at Linköping University Hospital. The participants with paroxysmal AF underwent time-resolved cardiac CT before catheter ablation. All participants were adults and the indication for ablation was according to the ESC guidelines for atrial fibrillation 2020 (4). They were all on anticoagulant therapy with NOACs. The exclusion criteria were previous cardiac surgery or ablation, uncontrolled hypertension (blood pressure >170/100 mmHg), more than moderate mitral valve regurgitation, more than moderate left ventricular dysfunction or dilation, and ongoing AF on the date of examination. One participant of this group was excluded because meshes could not be created for all steps in the CFD simulations. For this patient, the mesh elements got inverted at some point in the cardiac cycle, leading to negative volumes and the solver to stop. This was due to inaccuracies in the wall tracking, probably caused by a lack of contrast in the CT between the LAA and one pulmonary vein at some phases of the cardiac cycle. The participants without AF underwent clinical coronary CT angiography. For that group, the exclusion criteria were AF and more than mild valvular regurgitation. These participants were enrolled for a study that also analyzed 4D flow MRI data, thus contraindications to MRI were also an exclusion criterion (15). Further, 3 patients were removed because the CT angiography did not cover the full LA including the LAA. Additionally, one participant was removed because of diagnosis of AF 1 month after the examination.

The study was performed in agreement with the Declaration of Helsinki and had been approved by the local ethics board (Regionala etikprövningsnämnden i Linköping, 2018/275-31 and 2015/396-31). All study participants provided informed written consent for the study.

### CT data acquisition

Time-resolved CT images were acquired using a dual-source CT scanner (Somatom Force, Siemens Healthineers, Erlangen, Germany) with a single collimation width of 0.6 mm. The slice thickness was 0.5 mm with a 50% overlap for the control group, and 0.75 mm with a 50% overlap for the AF group. The pitch was 0.17–0.28 for the control group, and 0.15–0.26 for the AF group. The in-plane resolution ranged from 0.26 to 0.39 mm for the control group, and from 0.33 to 0.43 mm for the AF group. The images were reconstructed to 288–476 slices with 512*512 pixels. The CT exposure was 144–355 mAs for the control group, and 59–219 mAs for the AF group. The participants received an iodinated contrast medium to enhance the blood pool. The contrast volume was 55.5–72.3 ml at 4.7–6 ml/s in the control group, and 50–70 ml at 2.6–3.5 ml/s in the AF group. This was due to the different clinical questions in the two groups. In the control group, the focus was on the coronary arteries, while the focus was on the pulmonary veins and left atrium for the AF group. However, this did not influence the quality of contrast in the left atrium and left ventricle. The images were reconstructed to 20 timeframes, each representing 5% of the RR interval.

The LAA shape was categorized into one of four shapes [chicken wing, cauliflower, cactus, and windsock (7)] independently by two observers (S.B. and I.S.). In case of a disagreement, the LAA shape was reevaluated by both observers together until a consensus was reached. Further, the ostium plane was manually set for each patient in a 3D visualization of the LA and LAA blood pool by I.S. (EP cardiologist with more than 5 years of experience in cardiac imaging) and S.B. in agreement. It was set at the narrowest point of appendage diameter that could still be seen as a continuation of the general atrial wall.

### Segmentation and wall motion tracking

**Figure 1:**
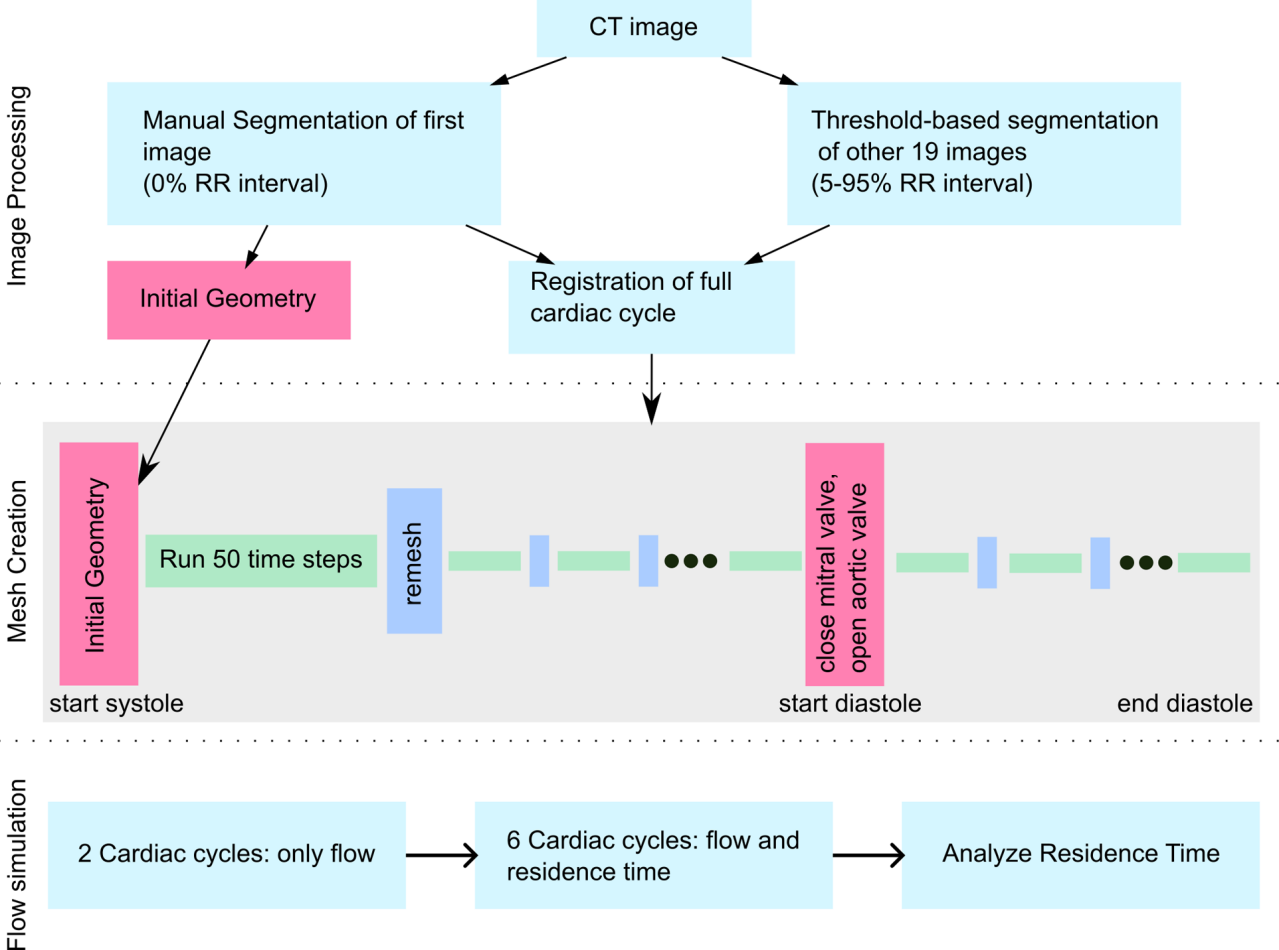
Workflow figure describing image processing, mesh creation and flow simulation.

The endocardial motion was tracked using a surface-based approach based on the time-resolved CT images. For this, the endocardium of the LA and LV was segmented using ITK-SNAP version 3.8.0 (www.itksnap.org) (27) at the end diastolic time frame (0% RR) and the geometry was further processed with Ansys SpaceClaim version 2019 R3 (Ansys Inc, Canonsburg, PA 15317 USA). Then, the endocardial surface was deformed using a non-rigid iterative closed point algorithm to match threshold-based segmentations of the other 19 cardiac phases, as described previously (14). The approach yields similar flow rates as those obtained by 4D flow MRI (14). The workflow of the process is visualized in Figure 1.

Using the deformation fields of the endocardial surface, morphological and functional cardiac descriptors were calculated, such as the minimal and maximal volume of the LA, LAA, and LV, as well as LV stroke volume, and peak flow rates through the mitral and aortic valves.

One of the main tasks of the LA is to act as a reservoir for blood leaving the lungs during ventricular systole and then delivering the blood to the ventricle during its filling. To evaluate atrial function the ratio of the maximum atrial volume to the ventricular stroke volume, further referred to as LA retention ratio, was calculated. This parameter indicates the maximum amount of blood that the atria can hold in relation to how much blood passes per heartbeat.

### CFD simulations

Blood stasis was assessed by computing the blood residence time derived from CFD simulations of the LA and LV, considering the participant-specific cardiac wall motion. The CFD simulations were performed using Ansys Fluent version 2019 R3, based on the process previously validated using 4D flow MRI (15). The motion of each boundary point was calculated by interpolating the motion of the 5 closest points in the registered geometry based on inverse distance weighting. To handle the extensive motion of the LA, LV, and complex wall structures, the mesh topology was adjusted every 50 time-steps, corresponding to 0.025 s. Meshes were refined close to the walls, with the wall edge length of 0.25 mm and a maximum edge length of 2 mm. The number of elements varied between the participants and during the cardiac cycle. The smallest mesh contained approximately 5 million cells and the largest mesh contained approximately 12 million elements. These settings were found to be sufficient after a mesh sensitivity study and were similar to our previous studies (15, 16). The time step was 5 × 10^–4^ s for all participants, based on previous studies (15, 28). The blood was modelled as a Newtonian fluid with a viscosity of 3.5 × 10^–3^ Pa s and a density of 1,060 kg/m^3^. The blood flow in the LA is laminar and hence no turbulent model was required (29). The cardiac model included the pulmonary veins, LA, LV, and left ventricular outflow tract. The pulmonary veins were cut around the first bifurcation, leading to 4–9 inflow openings, depending on the participant-specific geometry. The inflow openings, as well as the outflow tract at the aortic root were extended with straight pipes of ca. one-diameter length to ensure proper inflow characteristics. All openings were modelled as pressure openings, with the flow rate resulting from the motion of the endocardium. The heart valves were modelled as either open or closed for all participants.

The blood flow was initialized for 2 cardiac cycles, followed by 6 cardiac cycles solving an additional residence time equation [Eq. (1)] (30):

(1) $$\frac{\partial T_{\mathrm{res}}}{\partial t}+\frac{\partial }{\partial x_{i}}\left(u_{i}T_{\mathrm{res}}-D\frac{\partial T_{\mathrm{res}}}{\partial x_{i}}\right)=1$$

where $T_{\mathrm{res}}$ is the residence time; *t* is the time; $x_{i}$ are the spatial coordinates; $u_{i}$ is the velocity in each spatial direction; and *D* is the diffusivity, set to the blood diffusivity of 1.14 × 10^−11^ m^2^/s (31).

The residence time was evaluated after simulating the residence time for 6 cardiac cycles and the value converged. Each simulation was performed on 8 compute nodes, containing 2 Intel Xenon Gold CPUs, leading to 256 CPU cores per simulation. Simulation of one cardiac cycle took approximately 12 h.

### Statistical analysis

The inter-group comparisons were made using a two-sample *t*-test. To assess the association between the residence time and geometrical parameters, linear regression analysis was performed. All statistical computations were done using MATLAB R2021b and the significance level was set at 5%. Data is presented as means ± standard deviation unless stated otherwise.

## Results

### Study population

Table 1 shows characteristics of the individuals in the AF and controls groups. Participants in the AF group were on average 7 years younger than those in the non-AF group, but this difference is not statistically significant, and there were slightly fewer women in the AF group than in the control group. Further, the BMI was significantly higher in the non-AF group than that in the AF group. Four individuals in the AF group had mild mitral valve regurgitation. All other characteristics were not significantly different between the two groups.

**Table 1:** Participant characteristics.

	Controls (*n* = 8)	AF group (*n* = 21)	*p*-value
Age (years)	60 ± 14	67 ± 9	0.15
Women *n* (%)	3 (38)	6 (29)	0.84
Body surface area (m^2^)	2.13 ± 0.28	2.02 ± 0.17	0.22
Height (m)	1.73 ± 0.07	1.78 ± 0.09	0.17
Body mass index	32 ± 6	26 ± 2	**<0** **.** **001**
Heart rate	67 ± 7	62 ± 8	0.16
Diastolic blood pressure (mmHg)	139 ± 17	145 ± 20	0.47
Systolic blood pressure (mmHg)	76 ± 15	81 ± 12	0.37

Data are presented as mean ± standard deviation. The inter-group comparisons were made using a two-sample *t*-test, and *p*-values under 0.05 are marked in bold.

### Geometrical and functional parameters of the heart

We first analyzed several geometrical and functional parameters describing the LA and LV of controls and patients with AF. The mean values of these parameters are shown in Table 2. The LA and LAA volume indexed by the body-surface-area was significantly greater in patients with AF than the controls. The LA ejection fraction (EF) was significantly reduced in AF patients. The LAA ejection fraction also tended to be lower in AF patients, but this difference was not statistically significant. Parameters related to the LV such as the volume, ejection fraction, stroke volume, and peak flow rate through the aortic valve were not significantly different between the two groups. Further, in the control group, the left atrial retention ratio was 0.9, while it was 1.3 in the AF group. The difference in the left atrial retention ratio was statistically significant between the groups. Overall, the analysis showed that the two groups had different atrial geometry and function, while the ventricular size and function were comparable.

**Table 2:** Comparison of geometrical and functional parameters of controls and AF patients.

	Control (*n* = 8)	AF (*n* = 21)	*p*-value
LA Min Volume_,Idx_ (ml/m^2^)	19 ± 5	39 ± 14	**0.001**
LA Max Volume_,Idx_ (ml/m^2^)	41 ± 7	66 ± 15	**<0.001**
LA EF (%)	53 ± 7	43 ± 9	**0.007**
LAA Min Volume_,Idx_ (ml/m^2^)	0.9 ± 0.5	2.3 ± 1.3	**0.006**
LAA Max Volume_,Idx_ (ml/m^2^)	3.2 ± 1.3	6 ± 2.7	**0.009**
LAA EF (%)	72 ± 7	62 ± 12	0.052
LV End Systolic Volume_,Idx_ (ml/m^2^)	22 ± 11	26 ± 6	0.209
LV End Diastolic Volume_,Idx_ (ml/m^2^)	68 ± 18	78 ± 11	0.079
LV EF (%)	69 ± 6	67 ± 5	0.341
LV Stroke Volume (ml)	98 ± 26	106 ± 20	0.422
LA retention ratio (−)	0.9 ± 0.2	1.3 ± 0.3	**0** **.** **003**

Data are presented as mean ± SD; *p*-values were computed with two-sample *t*-test and *p*-values under 0.05 are marked in bold. EF, ejection fraction; Idx, indexed by the body surface area; LA, left atrium; LAA, left atrial appendage; LV, left ventricle.

### Left atrial blood residence time

**Figure 2:**
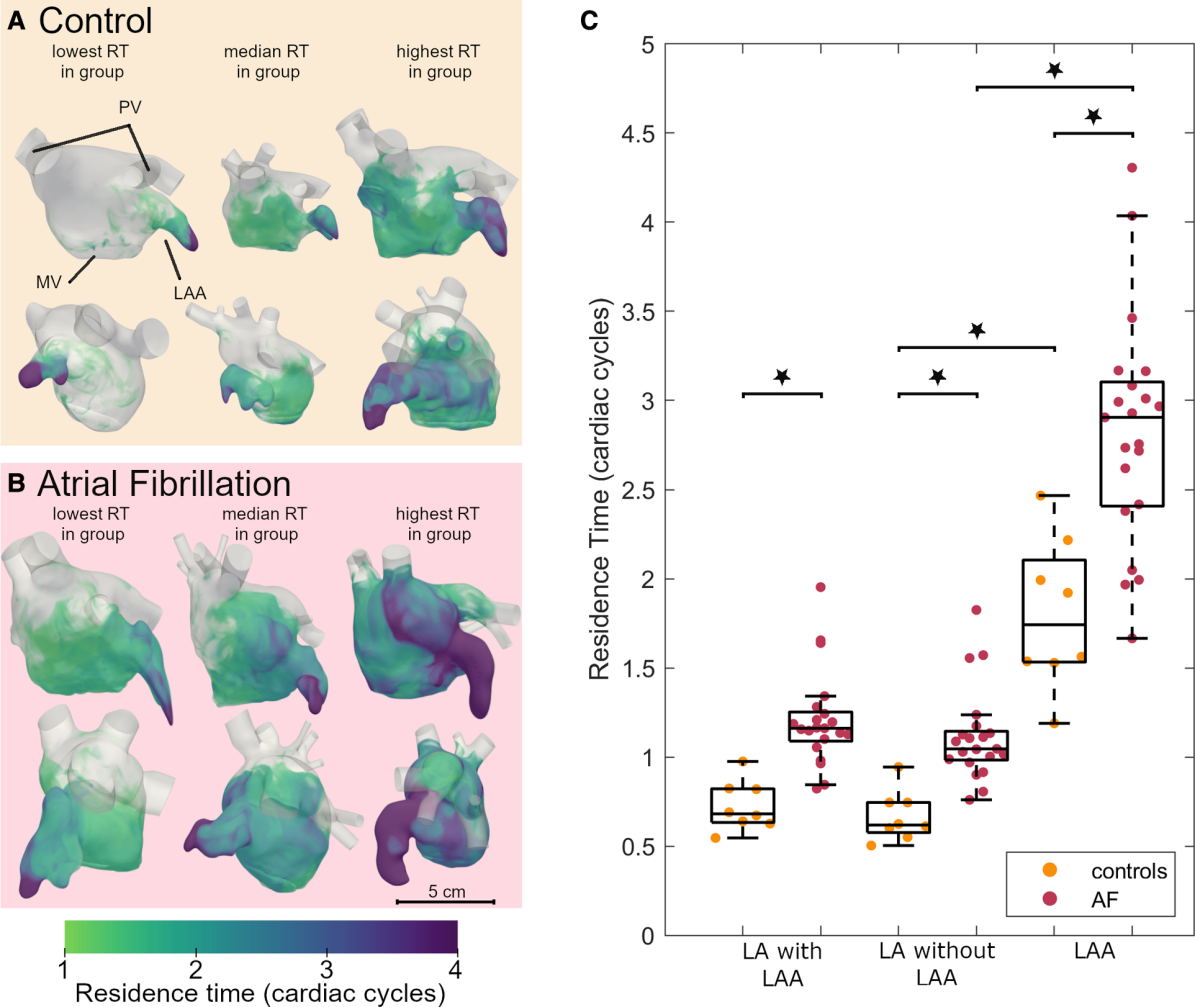
Residence time in the left atrium of AF patients and controls: volume renderings, each shown in two orthogonal views of 3 participants from the control (**A**) and AF groups (**B**). An animated version of this visualization can be found in the Supplementary Video. (**C**) Boxplot comparing the residence time after 6 cardiac cycles between controls and AF participants in the LA including the LAA, LA without the LAA and the LAA. Dots indicate actual data distribution. Star indicates significant difference with *p* < 0.001. The boxes indicated the 25th and 75th percentile and the central line indicates the median. The whiskers indicate the most extreme data point not considered an outlier. Inter-group comparisons were made using a two-sample *t*-test. AF, atrial fibrillation; LA, left atrium; LAA, left atrial appendage; MV, mitral valve; PV, pulmonary veins; RT, residence time.

Next, we evaluated the blood residence time in the LA derived from the CFD simulations, which is a marker for stasis. Volume renderings of the residence time at the end of systole for 3 representative participants from the AF and control groups each (participants with the lowest, median, and highest residence time in each group) are shown in the Figures 2A,B. The residence time distribution over the full cardiac cycle can be seen in the Supplementary Video. Blood resides the longest in the LAA for all participants, while the overall residence time is larger in the AF group than in the controls. The average residence time in the LA (excluding and including the LAA) and the LAA at the end of systole after six cardiac cycles is shown in Figure 2C. We noted a significantly greater residence time in both the LA and the LAA for AF patients compared to controls. The LAA residence time was significantly higher than the LA residence time in both groups.

### Relation between LA and LAA residence time and cardiac parameters

**Figure 3:**
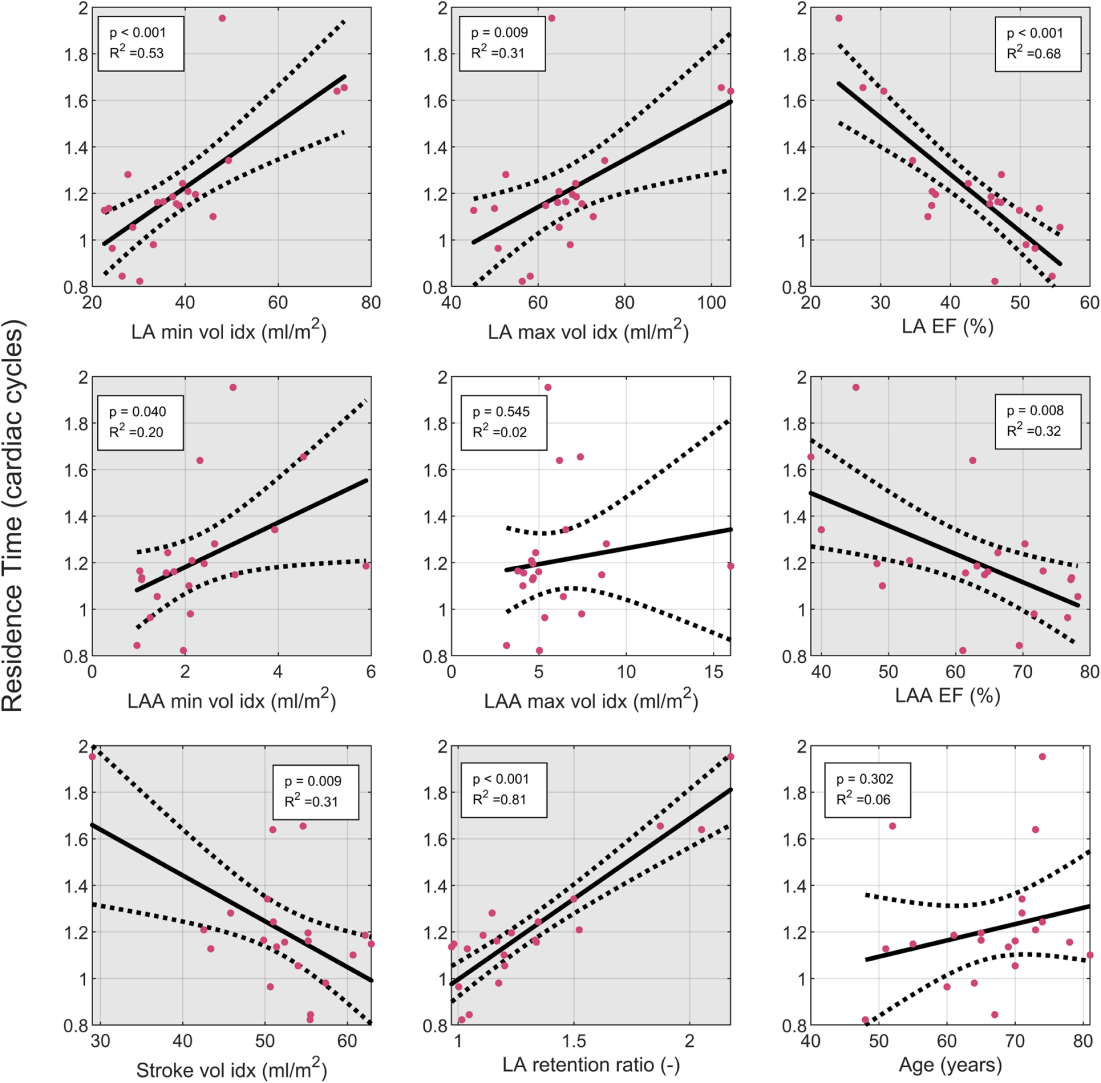
Linear regression analysis of the LA residence time: the residence time was evaluated in the LA plus LA. Solid line indicates fit from linear regression. Dashed lines indicate 95% confidence interval of linear regression. Grey background indicates significant correlation with *p* < 0.05. EF, ejection fraction; indexed: indexed by body surface area; LA, left atrium; LAA, left atrial appendage; LV, left ventricle, SV, stroke volume.

We then conducted univariate regression analysis between geometrical and function parameters and the residence time in the LA including the LAA in the AF group to identify parameters that can be related to an increased stasis (Figure 3). The residence time showed the strongest correlation with the LA retention ratio (*R* = 0.81, *p* < 0.001) and the LA EF (*R* = 0.68, *p* < 0.001), while the association to the LA volume was somewhat weaker. Parameters derived from the LAA were worse at predicting the residence time than parameters derived from the LA. There was a significant correlation between the ventricular stroke volume and the residence time, however, this association is strongly influenced by one data point with low stroke volume and high residence time. Excluding this point would remove the significant association.

It is debated whether the LAA morphology is associated with increased stasis in the LA. To explore this, we also assessed the relationship between LAA residence time and LAA morphology type. In patients with AF, we identified 16 LAAs as chicken wing type, 3 as cauliflower type, and 2 as windsock type. Because of the low number of non-chicken wing type LAAs, we analyzed them as one group. In the control group, the LAA in 7 participants was classified as chicken wing type and 1 as non-chicken wing type. Thus, the percentage of participants with chicken-wing type in the AF group (76%) was similar to the control group (88%, *p* = 0.52). In 7 AF patients and one control, the initial classification by 2 observers led to disagreement. The LAA type was then defined during a joint third analysis. There were no significant differences between the chicken wing and non-chicken wing type appendages in relation to the LAA residence time nor LA residence time in the AF group (Table 3). In the control group, only one participant was of non-chicken wing type, limiting the statistical reliability of a *t*-test comparison. However, the residence time values of this participant were within one standard deviation of the mean residence time of the chicken wing group. Thus, we could not find a relationship between LAA morphology and atrial residence time.

**Table 3:** LAA residence time by LAA type.

	Control CW (*n* = 7)	Control non-CW (*n* = 1)	AF CW (*n* = 16)	AF non-CW (*n* = 5)	*p*-value
RT LA with LAA (CC)	0.72 ± 0.14	0.67	1.24 ± 0.3	1.11 ± 0.18	0.38
RT LA without LAA (CC)	0.67 ± 0.14	0.62	1.13 ± 0.29	1.03 ± 0.16	0.46
RT LAA (CC)	1.8 ± 0.42	1.56	2.86 ± 0.67	2.72 ± 0.49	0.67

LAA, left atrial appendage; CW, chicken wing; AF, atrial fibrillation; RT, residence time; LA, left atrium; CC, cardiac cycles.

Finally, we did not detect any statistically significant differences between men and women in the residence time or geometrical or functional parameters of the LA and LAA (Supplementary Table S1).

## Discussion

In the current study, we characterized the hemodynamics, and key morphological and functional cardiac descriptors in AF patients by using CFD based on time-resolved CT data. We show that patients with paroxysmal AF in sinus rhythm have a significantly higher LA blood residence time than the control group. Further, the higher residence time was associated with a larger atrial volume, with an even stronger association between the residence time and the atrial ejection fraction, and the LA retention ratio.

The residence time, a marker for stasis, was increased in the AF group compared to controls, while the atrial size indexed by the body-surface area was larger and LA EF fraction was lower. This is in agreement with a large study with more than 13,000 participants, based on the UK biobank (32). This study identified an association between atrial fibrillation and increased atrial volumes and decreased LA EF. Further, they reported an association of decreased LA EF with stroke. Tan et al. found that increased atrial volumes are associated with stroke recurrence and diagnosis of AF in patients with stroke of undetermined source (33). Interestingly however, patients diagnosed with AF did not develop a second stroke, potentially due to successful anticoagulation. Thus, all recurrent strokes were not attributable to AF. Therefore, they discuss that atrial cardiomyopathy with diseased atrial wall and increased atrial volumes explained the risk of stroke. In agreement with this, we found that blood stays longer in the LA of AF patients during sinus rhythm. This increased stasis could be the reason for increased stroke risk in AF patients, even when in sinus rhythm, as well as why stroke risk is increased in patients with increased atrial volume, but no AF.

To better understand the relation between increased stasis and atrial remodeling, we conducted a linear regression analysis in the AF group. While the comparison of the AF patients with controls showed most prominent differences in the atrial volume, the linear regression identified LA EF and the LA retention ratio as the strongest parameters predicting stasis. This agrees with Raisi-Estabragh et al. (32), who found an association of LA EF with stroke, but not of atrial volume with stroke, in a multivariable linear regression model. Beside LA EF, we also found an association with the LA retention ratio. This parameter, combined with the LA EF is similar to the left atrial function index defined for ultrasound. Wong et al. found that the left atrial function index is associated with an increased risk of stroke in patients with coronary artery disease in the absence of AF, indicating that atrial dysfunction plays an important role for stroke risk (34). Our results are in line with the growing evidence that atrial cardiomyopathy rather than active fibrillation causes stroke. Our results highlight that besides increased inflammation, which has been discussed as the cause for this relationship, increased stasis in the left atrium, a key factor in Virchow's triad, could be central to the increased risk of stroke.

The LAA is thought to play a major role in thrombus formation, and we observed that the residence time was higher in the LAA than in the LA. However, the LAA ejection fraction was a weaker predictor of residence time than the LA ejection fraction. Further, LAA shape has been suggested as a biomarker for predicting stroke risk and a previous study reported a lower risk of stroke for the chicken wing LAA type (35). We did not find any correlation between the LAA shape and residence time in the current study. The analysis of LAA shape has been criticized as impractical and complicated (36). In addition, the reported prevalence of different LAA morphologies in AF varies between studies, e.g., Di Biase et al. (7) reported 26%–50% of AF patients with the chicken wing-type LAA, while we identified 79% participants as having the chicken wing-type LAA. Further, while Di Biase et al. (7) found a high interobserver agreement for the LAA morphological classification, classification of the LAA type based on their definitions can be challenging (36). While the LAA is the most likely region of the detection of thrombi, parameters describing the general atrial function rather than just the LAA seem to be better predictors for stroke risk. More research is needed to understand the complex relation between the atrium, LAA and stasis.

An explanation for the stronger association of the residence time with LAEF and LA retention ratio rather than the atrial volume is exemplified by the study patient with the highest residence time. This patient is a woman with a small ventricular volume in relation to her body surface area (End Diastolic Volume = 47 ml/m^2^; LV stroke vol = 29 ml/m^2^). The atrial geometry is shown in Figure 2B to the right. The patient's atrial volume in relation to the body surface area is close to the average of the group, while her atrial ejection fraction is the lowest in the patient group and LA retention ratio is the highest. For this individual, it appears important to relate the atrial size to the overall size of the heart to identify the increased stroke risk.

In this study, we used flow residence time as a marker for atrial stasis and potentially a predictor for stroke risk, similarly to (18, 21). Beside solving an additional scalar equation, stasis can also be estimated though a washout simulation, as done by (19), or by following particles (23). These blood-age-distribution approaches are similar to computing the residence time (21). Other studies use wall-shear-stress based measures to estimate the endothelial cell damage as a marker for thrombus formation (17, 22). Dueñas-Pamplona et al. compared blood-age-distribution based stasis measures with wall shear stress-based measures (21). In agreement with their finding, we found the highest residence time in the tip of the appendage. Dueñas-Pamplona et al. further found that wall shear stress-based measures, such as oscillatory shear index and the endothelial cell activation potential, also identified a region close to the LAA ostium. In our study, we chose to use residence time, a blood-age approach, as a marker for thrombus risk, as the wall structure in the LAA is highly trabeculated (37), to a degree that is not visible in time-resolved CT. Thus, close to the walls, CT based CFD simulations do not represent the flow conditions accurately. Therefore, we believe that markers that focus on the general flow field, rather than the flow close to the wall, are more reliable. However, it is not clear yet if stasis or endothelial cell damage, or a combination of both are the predominant mechanism for thrombus formation. Studies with more patients and stroke occurrence as an outcome are needed to properly identify the CFD markers that best predict stroke risk.

### Study limitations

The current study has some limitations. First, there were some differences in the baseline characteristics of the groups. The control group was not age-matched to the AF group (the control group participants were on average 7 years younger than the AF group participants). Thus, some differences between the groups could be linked to age rather than AF, especially since age is related to atrial enlargement (38). Furthermore, the control group participants were chosen from a group that had a clinical referral for cardiac CT, and could potentially have other underlying cardiac diseases, such as coronary stenosis. The body mass index in the control group was higher than that in the AF group, and the number of participants in the control group was smaller than the AF group. These factors could potentially weaken the statistical power of the comparison. In the future, studies with larger groups and better matching of underlying characteristics could better control for cofounding factors.

In addition, since the residence time was computed as a passive scalar that increases proportionally with time, longer simulation times lead to higher values for the residence time. According to previous studies, the LAA residence time reaches a steady state after 15–25 cardiac cycles (18, 20). However, already after 6 cardiac cycles, the residence time differs between patients in a similar way as the steady state solution. This indicates that analyzing the data after 6 cardiac cycles is sufficient for identifying differences between patients. However, since this numerical result is dependent on the number of cardiac cycles that were simulated, it might be difficult to compare values between different studies and further studies are needed to identify robust stasis parameters that do not require the simulation of many cardiac cycles. Further, in the current setup, the simulations require a long runtime and large computational power, as well as some manual adjustments. Before this technique can be used in a clinical setting, the run time needs to be optimized and manual steps automated, potentially using artificial intelligence.

Unfortunately, this study lacks follow-up data on stroke incidence, precluding the assessment of whether patients with prolonged residence time have an elevated stroke risk. Obtaining such data would be challenging due to the widespread prescription of anticoagulation medication, used to reduce the individual stroke risk. Thus, such a study would require a very large number of AF patients in order to include sufficient stroke incidences. Further, stroke occurrence in this patient group will primarily be caused by insufficient medical treatment. For example, patients with higher residence time might also be prescribed more potent anticoagulation therapy, due to other underlying conditions, and thus have a lower stroke risk after medication than a patient with medium residence time, but low or no anticoagulant treatment.

## Conclusions

CFD simulations of the left atrium are feasible in larger patient groups. With our CFD approach, we show that stasis is increased in AF patients even when they are investigated while in sinus rhythm, and is associated with atrial remodeling, which could possibly be the reason for increased stroke risk in the context of atrial cardiomyopathy.

## Data Availability

The original contributions presented in the study are included in the article/**Supplementary Material**, further inquiries can be directed to the corresponding author.
