# Corrigendum: Elevated atrial blood stasis in paroxysmal atrial fibrillation during sinus rhythm: a patient-specific computational fluid dynamics study

**DOI:** 10.3389/fcvm.2023.1301605

**Published:** 2023-10-09

**Authors:** Sophia Bäck, Iulia Skoda, Jonas Lantz, Lilian Henriksson, Lars O. Karlsson, Anders Persson, Carl-Johan Carlhäll, Tino Ebbers

**Affiliations:** ^1^ Unit of Cardiovascular Sciences, Department of Health, Medicine and Caring Sciences, Linköping University, Linköping, Sweden; ^2^ Center for Medical Image Science and Visualization (CMIV), Linköping University, Linköping, Sweden; ^3^ Department of Cardiology in Linköping, and Department of Health, Medicine and Caring Sciences, Linköping University, Linköping, Sweden; ^4^ Department of Radiology, and Department of Health, Medicine and Caring Sciences, Linköping University, Linköping, Sweden; ^5^ Department of Clinical Physiology in Linköping, and Department of Health, Medicine and Caring Sciences, Linköping University, Linköping, Sweden

**Keywords:** atrial fibrallation, computational fluid dynamics, left atrial appendage, computed tomgraphy, atrial cardiomyopathy, stroke

A Corrigendum on Elevated atrial blood stasis in paroxysmal atrial fibrillation during sinus rhythm: a patient-specific computational fluid dynamics study by Bäck S, Skoda I, Lantz J, Henriksson L, Karlsson LO, Persson A, Carlhäll C-J and Ebbers T. (2023) Front. Cardiovasc. Med. 10:1219021. doi: 10.3389/fcvm.2023.1219021

## Text correction

In the published article, there was an error. A cited study was wrongly summarized.

A correction has been made to the Introduction, paragraph 4. This sentence previously stated:

“However, both studies assumed the atrial walls to be rigid, hindering the analysis of the atrial function and its effects on stasis.”

The corrected sentence appears below:

“However, both studies did not take the patient specific wall motion into account, hindering the analysis of the atrial function and its effects on stasis.”

The authors apologize for this error and state that this does not change the scientific conclusions of the article in any way. The original article has been updated.

