# Supplementary material for: Elevated atrial blood stasis in paroxysmal atrial fibrillation during sinus rhythm: a patient-specific computational fluid dynamics study
Source: Front Cardiovasc Med. 2023 Aug 15;10:1219021. doi: 10.3389/fcvm.2023.1219021 (PMC10463733; doi:10.3389/fcvm.2023.1219021)
Supplement: Supplementary file 2 [file Table1.docx]

Supplementary Material

Patient-specific computational fluid dynamics reveals elevated blood stasis in paroxysmal atrial fibrillation during sinus rhythm

Sophia Bäck, Iulia Skoda, Jonas Lantz, Lilian Henriksson, Lars O. Karlsson, Anders Persson, Carl-Johan Carlhäll, Tino Ebbers*

* Correspondence: Tino Ebbers: tino.ebbers@liu.se

# Supplementary Table

Table S1: Statistical analysis of the differences between men and women in the current study. LA, left atrium; LAA, left atrial appendage; LV, left ventricle.

|  | *Women, all* | *Men, all* | *p, all* | *Women AF* | *Men AF* | *p, AF* | *Women control* | *Men control* | *p, control* |
| --- | --- | --- | --- | --- | --- | --- | --- | --- | --- |
| Residence Time LA + LAA | 1.15 | 1.07 | 0.53 | 1.35 | 1.21 | 0.26 | 0.79 | 0.76 | 0.73 |
| Residence Time LA | 1.06 | 0.99 | 0.56 | 1.24 | 1.11 | 0.30 | 0.74 | 0.70 | 0.74 |
| Residence Time LAA | 2.60 | 2.52 | 0.77 | 2.99 | 2.82 | 0.58 | 1.92 | 1.82 | 0.65 |
| LA Min Vol_,Idx_ (ml/m^2^) | 36 | 34 | 0.73 | 42 | 39 | 0.63 | 25 | 22 | 0.69 |
| LA Max Vol_,Idx_ (ml/m^2^) | 62 | 60 | 0.81 | 67 | 66 | 0.88 | 51 | 45 | 0.57 |
| LA EF (%) | 44 | 46 | 0.74 | 39 | 43 | 0.40 | 54 | 52 | 0.77 |
| LAA Min Vol_,Idx_ (ml/m^2^) | 2 | 2 | 0.97 | 2 | 2 | 0.82 | 1 | 1 | 0.83 |
| LAA Max Vol_,Idx_ (ml/m^2^) | 5 | 5 | 0.76 | 6 | 6 | 0.83 | 3 | 3 | 0.87 |
| LAA EF (%) | 63 | 65 | 0.61 | 56 | 62 | 0.51 | 70 | 71 | 0.80 |
| LV Min Vol_,Idx_ (ml/m^2^) | 22 | 25 | 0.34 | 24 | 26 | 0.43 | 19 | 21 | 0.70 |
| LV Max Vol_,Idx_ (ml/m^2^) | 69 | 75 | 0.20 | 70 | 78 | 0.15 | 66 | 67 | 0.88 |
| LV EF (%) | 68 | 68 | 0.75 | 66 | 67 | 0.93 | 72 | 70 | 0.68 |
| LV Stroke Volume (ml) | 90 | 103 | 0.06 | 89 | 106 | 0.07 | 91 | 98 | 0.58 |
| LA retention ratio (-) | 1.34 | 1.21 | 0.34 | 1.49 | 1.31 | 0.26 | 1.08 | 0.98 | 0.62 |
| Age (Years) | 67 | 65 | 0.47 | 69 | 66 | 0.52 | 64 | 61 | 0.62 |
| Body surface area (m^2^) | 1.92 | 2.05 | **0.05** | 1.91 | 2.02 | 0.12 | 1.94 | 2.12 | 0.25 |
| Height (m) | 1.69 | 1.77 | **0.01** | 1.69 | 1.78 | **0.03** | 1.69 | 1.73 | 0.27 |
| Body Mass index | 28 | 28 | 0.97 | 27 | 26 | 0.37 | 28 | 31 | 0.43 |
